# Supplementary material for: Detecting diagnostic features in MS/MS spectra of post-translationally modified peptides
Source: Nat Commun. 2023 Jul 12;14:4132. doi: 10.1038/s41467-023-39828-0 (PMC10338467; doi:10.1038/s41467-023-39828-0)
Supplement: Supplementary file 1 — Supplementary Information [file 41467_2023_39828_MOESM1_ESM.pdf]

Mining for ions: diagnostic feature detection in MS/MS spectra of post-translationally modified peptides

Daniel J. Geiszler[1], Daniel A. Polasky[2], Fengchao Yu[2], & Alexey I. Nesvizhskii[1,2,*]
[1]University of Michigan, Department of Computational Medicine and Bioinformatics
[2]University of Michigan, Department of Pathology

*Corresponding author: nesvi@med.umich.edu

Supplementary Figures:

Supplementary Figure 1: Increase in RNA crosslinked PSMs when including extra features identified by PTM-Shepherd.

Supplementary Figure 2: Histograms of fragment remainders across mass shifts.

Supplementary Figure 3: PTM-specific diagnostic ions in unmodified spectra.

Supplementary Figure 4: Trends in peptide remainder ions.

Supplementary Tables:

Supplementary Table 1: Diagnostic features for a cysteine probe.

Supplementary Table 2: Supplementary Table 2: PTM-Shepherd diagnostic features for RNA Xlinking data from offset search.

Supplementary Notes:

Supplementary Note 1: Analysis of ADP-Ribosylation ion specificity

Supplementary Note 2: Calculation of classification metrics from PTM-Shepherd

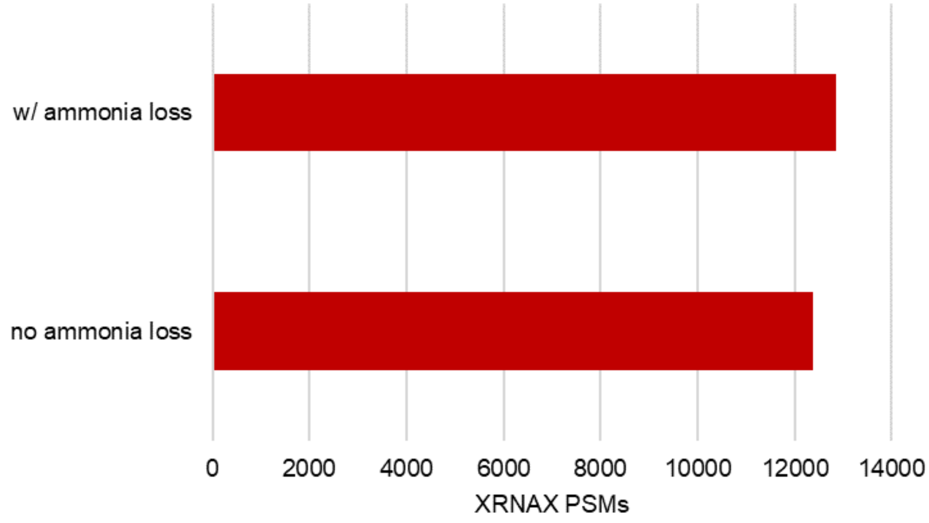

Supplementary Figure 1: Increase in RNA crosslinked PSMs when including extra features identified by PTM-Shepherd. Including the additional loss of ammonium detected only by PTM-Shepherd on top of the loss of ribose results in an extra 3.8% of crosslinked PSMs detected (12376 vs. 12848). Source data are provided as a Source Data file.

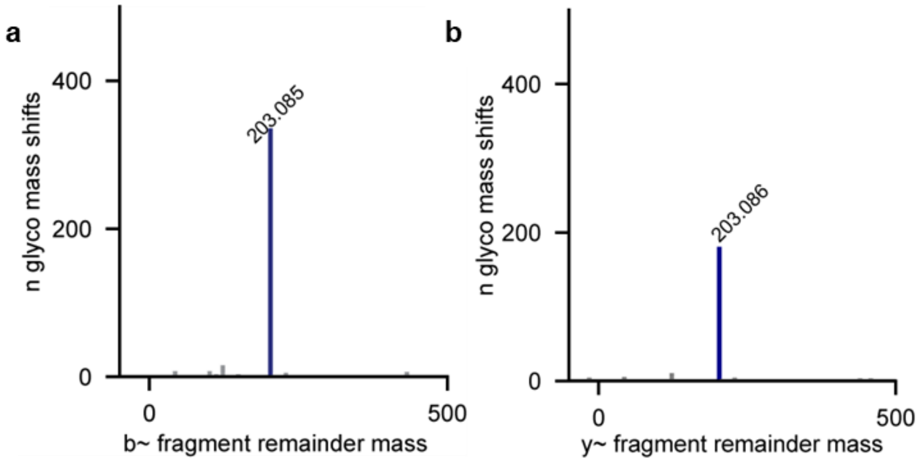

Supplementary Figure 2: Histograms of fragment remainders across mass shifts. (a) Fragments ions of *b*-type have a consistent and expected 203 Da remainder mass corresponding to a single HexNAc identified across many mass shifts. (b) Like *b*-ions, *y*-ions have a consistent and expected 203 Da remainder mass corresponding to a single HexNAc identified across many mass shifts. Source data are provided as a Source Data file.

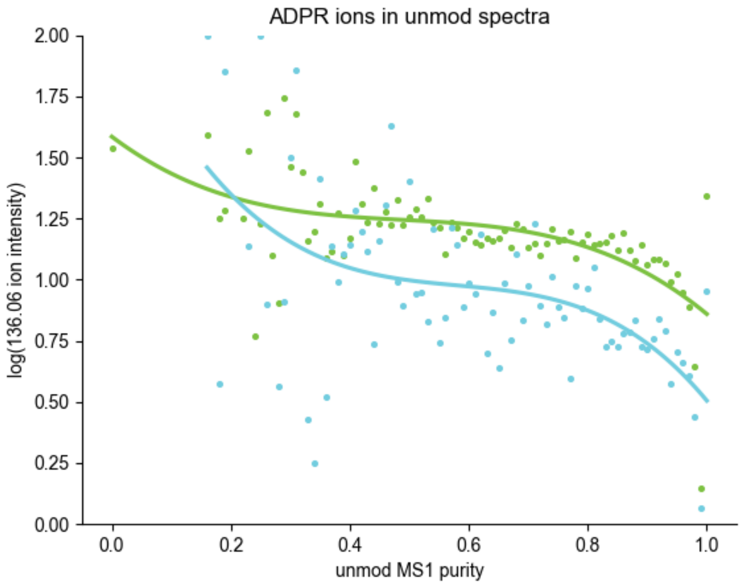

Supplementary Figure 3: PTM-specific diagnostic ions in unmodified spectra. As the purity of unmodified spectra decreases, the intensity of PTM-specific ions increases. Each datapoint represents the median of unmodified PSM spectral purities from 0.01 width purity bins to prevent overplotting at higher purities. Trendline was fit using Seaborn's regplot with order = 3. Purity was calculated using Philosopher. Source data are provided as a Source Data file.

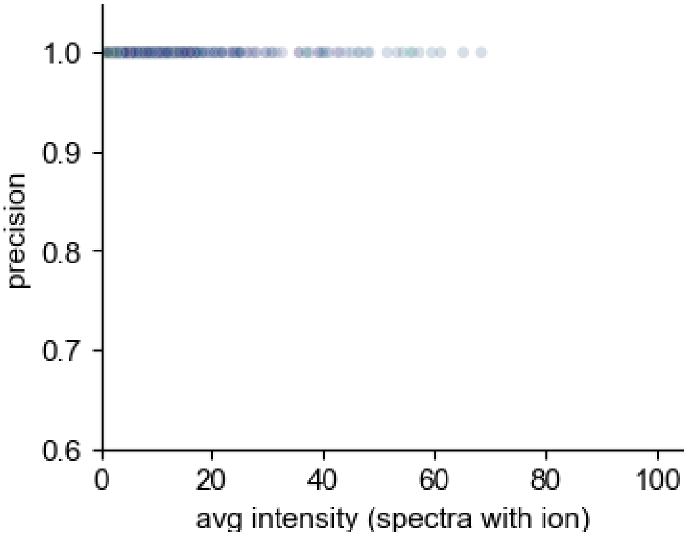

Supplementary Figure 4: Trends in peptide remainder ions. Unlike for diagnostic ions, peptide remainder ions retain their high precision due to being dependent on the mass of their host peptide and the lack of co-fragmentation interference. Source data are provided as a Source Data file.

peak apex	mod annotation	ion type	mass	delta mod. mass	remainder propensity	percent PSMs (mod)	percent PSMs (unmod)	avg. intensity (mod)	avg. intensity (unmod)	intensity fold change	auc
463.2364	Unannotated mass-shift 463.2364	diagnostic	301.1692			86.30	12.14	8.70	5.60	11.04	0.90
463.2364	Unannotated mass-shift 463.2364	diagnostic	327.185			88.80	20.32	8.83	5.76	6.70	0.89
463.2364	Unannotated mass-shift 463.2364	diagnostic	498.2314			98.80	51.98	31.51	14.87	4.03	0.89
463.2364	Unannotated mass-shift 463.2364	diagnostic	522.232			93.10	31.53	16.38	9.57	5.06	0.88
463.2364	Unannotated mass-shift 463.2364	diagnostic	424.2488			82.80	14.12	7.56	6.30	7.04	0.85
463.2364	Unannotated mass-shift 463.2364	diagnostic	227.084			85.40	22.3	5.81	4.34	5.12	0.85
463.2364	Unannotated mass-shift 463.2364	diagnostic	226.1012			45.70	2.51	2.34	1.97	21.60	0.72
463.2364	Unannotated mass-shift 463.2364	diagnostic	496.2164			46.10	7.39	3.60	6.65	3.38	0.69
463.2364	Unannotated mass-shift 463.2364	diagnostic	470.2254			40.40	3.17	2.64	6.70	5.03	0.68
463.2364	Unannotated mass-shift 463.2364	peptide	152.1038	-311.133		49.70	0.00	12.98	0.00	100.00	0.75
463.2364	Unannotated mass-shift 463.2364	peptide	180.1032	-283.133		47.50	0.00	13.94	0.00	100.00	0.74
463.2364	Unannotated mass-shift 463.2364	b	463.2364	0.00	44.11	48.50	0.53	33.32	28.37	100.00	0.74
463.2364	Unannotated mass-shift 463.2364	b	435.2420	-27.9944	30.39	27.20	0.26	15.19	11.03	100.00	0.63
463.2364	Unannotated mass-shift 463.2364	y	463.2364	0.00	26.65	48.40	0.26	33.23	30.72	100.00	0.74

Supplementary Table 1: Diagnostic features for a cysteine probe. Remainder propensity scores are present only for *b*- and *y*-remainder masses. Features corresponding to the set of modified or unmodified PSMs used in comparisons are labeled as (mod) and (unmod), respectively. The difference between the MS1 mass shift and the observed fragment remainder masses (i.e., the lost mass) is enumerated in the “delta mod mass” column.

peak apex	mod annotation	ion	mass	delta mod. mass	remainder propensity	percent PSMs (mod)	percent PSMs (unmod)	avg. intensity (mod)	avg. intensity (unmod)	intensity fold change
226.0594	Unannotated mass-shift 226.0594	diagnostic	133.0502			39	12.9	7.81	4.76	4.95
226.0594	Unannotated mass-shift 226.0594	diagnostic	115.0396			31.1	10.4	6.04	3.84	4.70
226.0594	Unannotated mass-shift 226.0594	b	94.0288	31.68	-132.031	36.9	1	47.65	24.91	70.60
226.0594	Unannotated mass-shift 226.0594	b	77.0052	18.47	-149.054	17.9	1	29.43	13.13	40.13
226.0594	Unannotated mass-shift 226.0594	y	94.0300	22.15	-132.029	35.7	1	46.95	24.91	67.30
226.0594	Unannotated mass-shift 226.0594	y	77.0068	15.11	-149.053	17.4	0.9	28.81	13.05	42.68
94.0170	Unannotated mass-shift 94.0170	diagnostic	215.0582			30.62	16.5	14.28	7.01	3.78
94.017	Unannotated mass-shift 94.0170	b	94.0170	36.73	0	39.53	0.9	38.37	26.18	64.38
94.017	Unannotated mass-shift 94.0170	b	77.0020	22.48	-17.015	21.71	1	34.51	12.29	60.95
94.017	Unannotated mass-shift 94.0170	b	-19.0644	20.81	-113.081	15.89	0.6	36.8	21.02	46.38
94.017	Unannotated mass-shift 94.0170	b	66.0272	20.08	-27.9898	15.89	1.3	19.82	23.58	10.28
94.017	Unannotated mass-shift 94.0170	y	94.0170	23.75	0	39.53	0.9	38	26.18	63.76
94.017	Unannotated mass-shift 94.0170	y	77.0064	14.41	-17.0106	19.38	0.9	30.34	13.05	50.05

Supplementary Table 2: PTM-Shepherd diagnostic features for RNA Xlinking data from offset search. Remainder propensity scores are present only for *b*- and *y*- remainder masses. Features corresponding to the set of modified or unmodified PSMs used in comparisons are labeled as (mod) and (unmod), respectively. The difference between the MS1 mass shift and the observed fragment remainder masses (i.e., the lost mass) is enumerated in the “delta mod mass” column.

Supplementary Note 1: Analysis of ADP-Ribosylation ion specificity. We attempted to do searches for ADP-Ribosylated (ADPR) peptides using parameters as close to FragPipe defaults as possible to make them reproducible for other users. The default ADPR workflow includes both a labile and variable modification search for ADPR, allowing competition between fragmented and intact forms of the modification. Because the zero bin is defined as peptides with no mass shift and PTMs identified as variable mods have no mass shift, some ADPR-containing peptides are also present in the unmodified peptide bin. However, these only account for roughly 1 in 7 PSMs in the unmodified bin, and as such cannot be driving the trends discussed here.

Supplementary Note 2: Calculation of classification metrics from PTM-Shepherd. PTM-Shepherd internally calculates a series of metrics to characterize diagnostic features. One of these is the Area Under the Curve of the Receiver Operating Characteristic Curve (AUC-ROC, or just AUC). This metric can optionally be added to PTM-Shepherd output tables. This metric can be computed directly from the U-statistic of the test group computed as part of the Mann-Whitney *U* test. The formula for this statistic is:

$$AUC_t = \frac{U_t}{n_t n_c} \quad (1)$$

where ***t*** and ***c*** stand for test and control groups and ***n*** is the respective groups sample sizes. This metric has a useful interpretation as a rank probability statistic, i.e., it can be directly interpreted as the probability that a randomly chosen value from group *t* will be higher a randomly chosen value from group *c*. Importantly, when calculated from the U-statistic, AUC is not sensitive to class imbalances. This allows comparisons between diagnostic ions for mass shifts that have different numbers of PSMs, as in Fig 5a-c. The second metric calculated in the manuscript is precision, also known as positive predictive power. In classification problems, this metric is the inverse of FDR. Whereas FDR can be interpreted as the probability that a hit is a false positive given that it is positive, precision can be interpreted as the probability that a hit is a true positive given that it is positive. When using the presence of a particular feature to classify whether the spectrum contains the PTM of interest, this can be calculated easily from PTM-Shepherd output by the equation:

$$precision = \frac{proportion_m}{proportion_u + proportion_m} \quad (2)$$

where *u* and *m* correspond to unmodified and modified PSMs and proportion is the proportion of spectra containing the ion. Again, this metric is not sensitive to class imbalances when calculated this way, enabling direct comparisons between mass shifts with different numbers of PSMs.
