# Supplementary material for: Detecting diagnostic features in MS/MS spectra of post-translationally modified peptides
Source: Nat Commun. 2023 Jul 12;14:4132. doi: 10.1038/s41467-023-39828-0 (PMC10338467; doi:10.1038/s41467-023-39828-0)
Supplement: Supplementary file 8 — Reporting Summary [file 41467_2023_39828_MOESM8_ESM.pdf]

nature portfolio

Corresponding author(s): Alexey I. Nesvizhskii

Last updated by author(s): Jun 19, 2023

Reporting Summary

Nature Portfolio wishes to improve the reproducibility of the work that we publish. This form provides structure for consistency and transparency in reporting. For further information on Nature Portfolio policies, see our Editorial Policies and the Editorial Policy Checklist.

Statistics

For all statistical analyses, confirm that the following items are present in the figure legend, table legend, main text, or Methods section.

n/a	Confirmed	
x		The exact sample size (*n*) for each experimental group/condition, given as a discrete number and unit of measurement
x		A statement on whether measurements were taken from distinct samples or whether the same sample was measured repeatedly
	x	The statistical test(s) used AND whether they are one- or two-sided<br>*Only common tests should be described solely by name; describe more complex techniques in the Methods section.*
x		A description of all covariates tested
	x	A description of any assumptions or corrections, such as tests of normality and adjustment for multiple comparisons
x		A full description of the statistical parameters including central tendency (e.g. means) or other basic estimates (e.g. regression coefficient) AND variation (e.g. standard deviation) or associated estimates of uncertainty (e.g. confidence intervals)
	x	For null hypothesis testing, the test statistic (e.g. *F*, *t*, *r*) with confidence intervals, effect sizes, degrees of freedom and *P* value noted<br>*Give P values as exact values whenever suitable.*
x		For Bayesian analysis, information on the choice of priors and Markov chain Monte Carlo settings
x		For hierarchical and complex designs, identification of the appropriate level for tests and full reporting of outcomes
	x	Estimates of effect sizes (e.g. Cohen's *d*, Pearson's *r*), indicating how they were calculated

Our web collection on statistics for biologists contains articles on many of the points above.

Software and code

Policy information about availability of computer code

Data collection	No data was collected.
Data analysis	Tools used in this study: FragPipe v18.0, MSFragger v3.5, Philosopher v4.2.2, PTM-Shepherd v2.0.0, Proteowizard v3.0.11392, Proteowizard v3.0.19296. PTM-Shepherd v2.0.0 can be accessed at https://github.com/Nesvilab/PTM-Shepherd/releases/tag/v2.0.0. Secondary analysis was perform using Python 3.7 with Numpy v1.21.2, Seaborn v0.11.2, Pandas v1.1.5, and sklearn v1.0. Custom Python scripts can be accessed at https://doi.org/10.5281/zenodo.8056053.

For manuscripts utilizing custom algorithms or software that are central to the research but not yet described in published literature, software must be made available to editors and reviewers. We strongly encourage code deposition in a community repository (e.g. GitHub). See the Nature Portfolio guidelines for submitting code & software for further information.

Data

Policy information about availability of data

All manuscripts must include a data availability statement. This statement should provide the following information, where applicable:

- Accession codes, unique identifiers, or web links for publicly available datasets
- A description of any restrictions on data availability
- For clinical datasets or third party data, please ensure that the statement adheres to our policy

All data used in this manuscript is publicly available and previously published. The following datasets were downloaded from ProteomeXchange: the cysteine probes data are available under the access code PXD028853, RNA crosslink data are available under accession code PXD023401, and human brain proteome data are

available under accession code PXD010154. IMAC-enriched glycan datasets were downloaded from the CPTAC data portal. Source data are provided with this paper. Diagnostic ion mining output from the cysteine probe dataset is available as a Supplementary Table. Output associated with the RNA crosslink data analyzed throughout this paper is available as a Supplementary Table and Supplementary Data. Output associated with the human brain proteome and IMAC-enriched glycopeptides are available as Supplemental Data or Supplemental Tables. All data was searched against the Uniprot reviewed protein sequences database retrieved on 13 June 2021.

Human research participants

Policy information about studies involving human research participants and Sex and Gender in Research.

Reporting on sex and gender	NA
Population characteristics	NA
Recruitment	NA
Ethics oversight	NA

Note that full information on the approval of the study protocol must also be provided in the manuscript.

Field-specific reporting

Please select the one below that is the best fit for your research. If you are not sure, read the appropriate sections before making your selection.

[x] Life sciences [ ] Behavioural & social sciences [ ] Ecological, evolutionary & environmental sciences

For a reference copy of the document with all sections, see nature.com/documents/nr-reporting-summary-flat.pdf

Life sciences study design

All studies must disclose on these points even when the disclosure is negative.

Sample size	We selected 5 datasets to use in this study: one to show that we could reproduce existing results from previous versions that we experimentally validated, one to show that we did not produce extraneous false positive results as a negative control, and three applications. The first two datasets showed that our method is statistically rigorous, identifying true positives while controlling false hits, and were sufficient for the analysis. The latter three cover areas that are of interest to proteomics researchers and were selected to increase the impact of the publication by appealing to researchers in chemoproteomics, glycoproteomics, proteogenomics, and generic labile PTMs.
Data exclusions	All data used in this study is available in the supplemental data. No data was excluded.
Replication	Our paper begins by recapitulating results for a prior version of the tool that was experimentally validated, thus proving reproducibility. All data is publicly available and tools can be downloaded from https://fragpipe.nesvilab.org/.
Randomization	Only one instance of randomization was required: searching of the experimentally constructed negative control database. We randomized mass shifts within the database to show that our method does not produce erroneous false positives in datasets where there should be none. Randomization was not relevant to the other studies in the manuscript because no other studies had data that required equal, unbiased treatment between multiple groups.
Blinding	Blinding is not relevant to our study because it is not a testing of specific treatment conditions.

Reporting for specific materials, systems and methods

We require information from authors about some types of materials, experimental systems and methods used in many studies. Here, indicate whether each material, system or method listed is relevant to your study. If you are not sure if a list item applies to your research, read the appropriate section before selecting a response.

Materials & experimental systems

n/a	Involved in the study
☒	☐ Antibodies
☒	☐ Eukaryotic cell lines
☒	☐ Palaeontology and archaeology
☒	☐ Animals and other organisms
☒	☐ Clinical data
☒	☐ Dual use research of concern

Methods

n/a	Involved in the study
☒	☐ ChIP-seq
☒	☐ Flow cytometry
☒	☐ MRI-based neuroimaging
